# Effect of Metabolizable Protein Supply on Milk Performance, Ruminal Fermentation, Apparent Total-Tract Digestibility, Energy and Nitrogen Utilization, and Enteric Methane Production of Ayrshire and Holstein Cows

**DOI:** 10.3390/ani13050832

**Published:** 2023-02-24

**Authors:** Chaouki Benchaar, Fadi Hassanat, Karen A. Beauchemin, Daniel R. Ouellet, Hélène Lapierre, Cristiano Côrtes

**Affiliations:** 1 Agriculture and Agri-Food Canada, Sherbrooke Research and Development Centre, Sherbrooke, QC J1M 0C8, Canada; 2 Agriculture and Agri-Food Canada, Quebec Research and Development Centre, Québec, QC G1V 2J3, Canada; 3 Agriculture and Agri-Food Canada, Lethbridge Research and Development Centre, Lethbridge, AB T1J 4B1, Canada; 4 Agrinova, 640 rue Côté, Alma, QC G8B 7S8, Canada

**Keywords:** metabolizable protein, Ayrshire, Holstein, milk production, enteric methane, N efficiency

## Abstract

**Simple Summary:**

The effect of increasing metabolizable protein supply (85%, 100%, and 115% of requirement) on milk production, energy and nitrogen (N) utilization, and enteric methane (CH_4_) emissions of Ayrshire and Holstein cows was investigated in this study. Although Ayrshire cows consumed less dry matter and produced less milk than Holstein cows, feed efficiency (kg energy-corrected milk/kg of dry matter intake), CH_4_ emission intensity (g/kg milk), and urinary N excretion (g/100 g N intake) did not differ between the two breeds. Energy-corrected milk increased and N use efficiency (g N milk/100 g N intake) decreased with increasing metabolizable protein supply, whereas CH_4_ emissions were unaffected by metabolizable protein supply.

**Abstract:**

In North America, the nutrient requirements of dairy cattle are predicted using the Cornell Net Carbohydrate and Protein System (CNCPS) or the National Research Council (NRC). As Holstein is the most predominant dairy cattle breed, these models were developed based on the phenotypic, physiological, and genetic characteristics of this breed. However, these models may not be appropriate to predict the nutrient requirements of other breeds, such as Ayrshire, that are phenotypically and genetically different from Holstein. The objective of this study was to evaluate the effects of increasing the metabolizable protein (MP) supply using CNCPS on milk performance, ruminal fermentation, apparent total-tract digestibility, energy and N utilization, and enteric methane production in Ayrshire vs. Holstein lactating dairy cows. Eighteen (nine Ayrshire; nine Holstein) lactating cows were used in a replicated 3 × 3 Latin square design (35-d periods) and fed diets formulated to meet 85%, 100%, or 115% of MP daily requirement. Except for milk production, no breed × MP supply interaction was observed for the response variables. Dry matter intake (DMI) and the yields of energy-corrected milk (ECM), fat, and protein were less (*p* < 0.01) in Ayrshire vs. Holstein cows. However, feed efficiency and N use efficiency for milk production did not differ between the two breeds, averaging 1.75 kg ECM/kg DMI and 33.7 g milk N/100 g N intake, respectively. Methane yield and intensity and urinary N also did not differ between the two breeds, averaging 18.8 g CH_4_ /kg DMI, 10.8 g CH_4_ /kg ECM, and 27.6 g N/100 g N intake, respectively. Yields of ECM and milk protein increased (*p* ≤ 0.01) with increasing MP supply from 85% to 100% but no or small increases occurred when MP supply increased from 100 to 115%. Feed efficiency increased linearly with an increasing MP supply. Nitrogen use efficiency (g N milk/100g N intake) decreased linearly (by up to 5.4 percentage units, (*p* < 0.01) whereas urinary N excretion (g/d or g/100 g N intake) increased linearly (*p* < 0.01) with an increasing MP supply. Methane yield and emission intensity were not affected by MP supply. This study shows that feed efficiency, N use efficiency, CH_4_ (yield and intensity), and urinary N losses did not differ between Ayrshire and Holstein cows. Energy-corrected milk yield and feed efficiency increased, but N use efficiency decreased and urinary N losses increased with increasing dietary MP supply regardless of breed. Ayrshire and Holstein breeds responded similarly to increasing MP levels in the diet.

## 1. Introduction

Ayrshire cows represent 1.5% and 0.3% of the dairy cow population in Canada [1] and USA [2], respectively, as the overwhelming majority (i.e., 94%) of dairy cows are Holstein. Ayrshire is a smaller breed than Holstein (600 vs. 700 kg mature body weight [BW]) with lower milk production, but higher milk fat and milk protein concentrations, averaging 25.0 vs. 33.5 kg/d, 4.09 vs. 3.96 %, and 3.39 vs. 3.27%, respectively [3,4,5]. Ayrshire cows are known for the good quality of the udder, which may help in reducing the incidence of mastitis and lower milk somatic cell count (SCC). In addition, they are subject to less leg and feet problems, and are highly adaptable to various management systems and to harsh conditions [6,7]. In addition, the replacement rate of Ayrshire cows is lower than other dairy cattle populations in Canada (38 vs. 41%, [1]), which represents an advantage over other breeds such as Holstein. Distinctive phenotypic and genetic characteristics of Ayrshire cows may thus result in different nutritional requirements compared with Holstein cows.

The nutritional requirements of Ayrshire cows are not well defined, as limited research has been conducted on their nutrient requirements. As Holstein is the most predominant dairy cattle breed, the phenotypic, physiological, and genetic characteristics of Holsteins have been used to develop ration formulation programs. However, other breeds (such as Ayrshire) may have different digestive physiology and ruminal microbial diversity, affecting their nutrient requirements and capacity to digest feeds. For instance, a number of studies have indicated significant variation between Holstein and Jersey cows in gastro-intestinal tract size, the passage rate of digesta, the type and abundance of ruminal microbes, and the concentration and pattern of volatile fatty acid (VFA) [8,9,10,11]. Welch et al. [8] reported variation between cattle breeds in rumination time. Rodriguez et al. [9] and Olijhoek et al. [11] reported higher concentrations of ruminal fermentation end-products (total VFA and ammonia [NH_3_]) and greater organic matter (OM), neutral detergent fiber (NDF), and starch digestibility in Jersey compared with Holstein cows fed the same diet, suggesting higher microbial activity and digestive capacity in the gastro-intestinal tracts of Jersey cows. Beecher et al. [10] reported bigger reticulo-rumen and omasum per unit of BW in Jersey cows compared with Holstein cows and variation in ruminal microbial populations between the two breeds. These differences may lead to variations in voluntary DMI potential, the ability to digest fiber, and energy and protein (i.e., MP) requirements for milk production. For instance, Rodriguez et al. [9] reported different responses to dietary rumen undegradable protein (RUP) levels between Holstein and Jersey cows. Such differences may as well exist between Ayrshire and Holstein cows. To the best of our knowledge, very few studies have been conducted to determine differences between Ayrshire and Holstein cows in terms of their energy and N metabolism [12,13].

Nitrogen metabolism in the rumen and the ability to convert rumen degradable protein (RDP) to microbial crude protein is largely influenced by DMI, the degradation rate of feed in the rumen, the rate of passage through the gastro-intestinal tract, and the function of ruminal microorganisms [14]. Protein requirement can be defined as the amount of MP supply required to maximize milk yield. It could be also defined as the amount of RDP supply required to maximize microbial protein synthesis and fiber digestibility. Dairy cows require a given amount of amino acids (AA) absorbed at the intestinal level to meet their protein requirements (i.e., MP) for maintenance, pregnancy, and targeted levels of milk production [14,15]. Rations are formulated to provide minimal RDP to optimize fiber digestion and to maximize microbial protein synthesis [14], which is estimated to provide 52–63% of MP requirements of high producing cows [16,17]. Sources of RUP are then added in the diet to meet the estimated MP requirement at 100%.

Currently, ration formulation for Ayrshire dairy cows is performed using models based on the nutrient requirements for the Holstein breed and by taking into account differences in BW, predicted DMI, milk production, and milk composition. When the MP supply is inadequate, milk production decreases and the genetic potential of dairy cows to produce milk is not achieved. On the other hand, when the MP supply is above the requirement, N milk efficiency declines and urinary N excretion increases, which adversely affect the environmental footprint of milk production [18,19]. One of these two scenarios is likely to occur when the MP requirements of minor breeds of dairy cows, such as Ayrshire, are not well defined. In the present study, we hypothesized that given the differences in phenotypic and the genetic characteristics of Ayrshire versus Holstein cows, the MP requirements of Ayrshire cows to optimize milk production and feed efficiency may differ from that of Holstein cows, and that ration formulation using diet models [e.g., Cornell Net Carbohydrate and Protein System (CNCPS; version 6.55)] would not accurately predict the MP requirements of the Ayrshire breed.

The environmental footprint of milk production also encompasses enteric CH_4_ emissions. Potential variation between Ayrshire and Holstein cows in DMI (due to differences in BW), milk production, ruminal microbial populations, and VFA pattern can influence CH_4_ emission yield (g/kg DMI) and intensity (g/kg ECM). To our knowledge, studies on enteric CH_4_ emissions from Ayrshire cows are scarce [20,21] and none of the existing studies compared emissions from Ayrshire cows versus Holstein cows. We hypothesized that differences between the two breeds in the size of the gastro-intestinal tract, ingestion capacity, and ruminal feed degradation (rate, passage) may result in different CH_4_ emissions. Thus, the objective of this study was to investigate the effect of dietary MP supply on the milk production, milk composition, nutrient utilization, ruminal fermentation characteristics, apparent total-tract digestibility, and enteric CH_4_ production of Ayrshire and Holstein dairy cows.

## 2. Materials and Methods

This study was conducted at the Sherbrooke Research and Development Centre, (Sherbrooke, QC, Canada). Animal procedures were conducted under the approval of the local Institutional Animal Care Committee (number 566) and in accordance with the guidelines of the Canadian Council on Animal Care [22].

### 2.1. Cows, Experimental Design, and Diets

Eighteen (9 Ayrshire; 9 Holstein) multiparous lactating cows, of which 6 of each breed were ruminally cannulated (10 cm, Bar Diamond Inc., Parma, ID, USA), were used in a replicated 3 × 3 Latin square design (35-d periods). The 2 breeds were offered diets balanced to supply 85%, 100%, or 115% of the daily MP requirement. Experimental diets (Table 1) were formulated using the CNCPS model (version 6.55).

Prior the start of the experiment, Holstein and Ayrshire cows were kept in tie-stall and fed (ad-libitum) diets (pre-experiment diets) as a total mixed ration formulated (CNCPS, version 6.55) to meet net energy of lactation (NE_L_) and their MP requirements (i.e., 100% supply). Differences in BW, DMI, milk production, and milk composition between Ayrshire and Holstein cows required balancing breed-specific diets to meet their nutrient requirements. When fed the pre-experiment diets, the Holstein cows averaged (mean ± SD) 26.0 ± 2.34 kg/d DMI, 46.3 ± 4.5 kg/d milk, 3.96 ± 0.38% milk fat, and 2.97 ± 0.14% milk protein. The Ayrshire cows averaged 21.6 ± 1.58 kg/d DMI, 35.7 ± 3.4 kg/d milk, 4.18 ± 0.35% milk fat, and 3.28 ± 0.16% milk protein. In terms of absolute values (i.e., amount of MP supplied), the diets supplied 2710 and 2429 g/d of MP for Holstein and Ayrshire cows, respectively. Based on these data and the characteristics of the cows at the start of the trial (Holstein: 92 ± 22 days in milk (DIM), 701 ± 55.8 kg BW; Ayrshire: 99 ± 16 DIM; 566 ± 48.5 kg BW), experimental diets were balanced to supply 85%, 100%, or 115% of the daily MP requirement (Table 1). During the experiment, cows were housed in individual tie-stalls and had free access to water. The diets were delivered in equal meals twice daily (0930 and 2130 h). One Holstein cow was diagnosed with severe foot lesions causing lameness and was eliminated from the experiment.

The experimental diets were offered to cows to be fed ad libitum (5% orts, on an as-fed basis) as a total mixed ration with forage:concentrate ratio (on a DM basis) of 67:33 for Holstein cows and 62:38 for Ayrshire cows. For each breed, dietary forage proportions (i.e., corn silage; alfalfa silage; timothy hay) were maintained constant across MP supply levels. Increasing MP supply from 85% to 100% of the requirements was achieved by decreasing urea, corn grain, and soybean hulls proportions while increasing soybean meal, heat-treated soybean meal, and corn gluten meal proportions. Increasing MP supply from 100% to 115% was achieved by decreasing corn grain, soybean hulls, and soybean meal proportions, while increasing heat-treated soybean meal and corn gluten meal proportions. Ingredient and chemical composition of the experimental diets are detailed in Table 1.

Feed intake, milk production, milk composition, and BW were determined for 18 cows (i.e., 6 Latin squares) whereas the 12 ruminally cannulated cows (i.e., 4 Latin squares) were used for measurement of enteric CH_4_ production, ruminal fermentation characteristics, apparent total-tract digestibility, and N excretion. The availability of only 2 chambers (1 cow/chamber) to determine enteric CH_4_ production of 12 cows (3 cows/Latin square) necessitated staggering the Latin squares by 5 d and CH_4_ production measurements by 5 d. For each Latin square, the 35-d (including 5 d of staggering) were organized as follows: adaptation to dietary treatments from d 1 to d 14; CH_4_ production from d 15 to d 23; ruminal fluid sampling from d 25 to d 26; feed intake, milk production, milk composition, apparent total-tract digestibility and N excretion from d 27 to d 33; and BW on d 34 and d 35.

### 2.2. Apparent Total-Tract Digestibility, N Excretion, and BW

Intake, apparent total-tract digestibility, and N excretion were measured as described by Benchaar [23]. Feed consumption was recorded daily by weighing feeds offered to and refused by the cows. Samples of the experimental diets, feed ingredients, refusals, and feces were collected daily and stored at −20 °C. These samples were later thawed, composited by cow within period, freeze-dried, ground to pass a 1-mm screen using a Wiley mill (standard model 4; Arthur M. Thomas, Philadelphia, PA, USA) and analyzed for DM, OM, total N, neutral detergent fiber (NDF), acid detergent fiber (ADF), starch, and gross energy (GE). Total collection of feces and urine was performed as described by Benchaar [23] by fitting cows with harnesses and tubes allowing the collection of feces and urine separately. A representative (2%) sample of thoroughly mixed feces was collected daily. Urine was collected in containers containing appropriate amounts of H_2_SO_4_ to keep pH < 2.0. A representative urine sample (2%) was collected and kept frozen at −20 °C until analyzed for total N and purine derivatives (allantoin and uric acid). Cows were weighed at the beginning and the end of each experimental period on 2 consecutive days before the a.m. feeding and after the a.m. milking.

### 2.3. Ruminal Fermentation Characteristics and Protozoa Enumeration

Ruminal fluid was collected from each cow before (0 h) and 1, 2, 4, 6, and 8 h after the a.m. meal over 2 days as described in Benchaar et al. [24]. A total of 250 mL was collected from several locations (i.e., anterior dorsal, anterior ventral, medium ventral, posterior dorsal, and posterior ventral) within the rumen using a 50-mL syringe attached to a stainless tube ending with a probe covered by a fine metal mesh (RT Rumen Fluid Collection Tube, Bar Diamond Inc., Parma, ID, USA). Ruminal fluid was subsampled (15 mL) and frozen immediately at −20 °C for later determination of VFA concentrations. Additional subsamples (15 mL) were acidified to pH 2 with 50% sulfuric acid and frozen at –20 °C for later determination of NH_3_ concentrations.

Protozoa were counted from ruminal content collected before (0 h) and 4 h after the a.m. feeding as described in Benchaar et al. [24]. Ruminal content (approximately 1 L) was squeezed through 4 layers of cheesecloth and a 5-mL portion of the squeezed ruminal fluid was preserved using 5 mL of methyl green formalin-saline solution for protozoa enumeration [25]. Protozoa samples were stored at room temperature in darkness until counting. Protozoa were microscopically enumerated using a counting chamber (Neubauer Improved Bright-Line counting cell, 0.1 mm depth; Hausser Sc0.82ientific, Horshamm, PA, USA).

Ruminal pH was measured continuously on 12 cows for 48 h using the LRCpH bolus system (DASCOR Inc., Oceanside, CA, USA) initially described in Penner et al. [26]. The system consisted of a battery-powered LRCpH data logger connected to LRpH sensors with ceramic frit, enveloped by polyvinyl chlorine case. The sensors were calibrated to pH 4.00 and 7.00 at 39 °C prior to utilization. The probes were attached to 1.5-kg metal weights to ensure that they were always immersed in the ruminal fluid and then placed in the ventral sac of the rumen of each cow. Ruminal pH was recorded every 2 min. After 48 h, the probes were removed and data were downloaded. The probes were then immersed in pH 4.00 and pH 7.00 buffers kept at 39 °C, and the probes measurement drifted positively at an average of 2.03 ± 1.30%. Data were processed with DASCOR LRCpH software V-6.1.0. and were corrected for drift using a linear equation of the initial and final standard measurements from each cow/period. The pH data were summarized as mean pH, minimum pH, maximum pH, time spent below pH 6.0, time spent below pH 5.6, and time spent below pH 5.2 [27,28].

### 2.4. Milk Production and Milk Composition

Cows were milked in their stalls twice daily at 7:00 and 19:00, and milk production was recorded at each milking. The data recorded during the 7 d of apparent total-tract digestibility measurement were used for the statistical analysis. During that period, milk samples were taken from each cow at each milking, stored at 4 °C with a preservative (2-bromo-2-nitropropan-1,3-diol), and sent to a commercial laboratory (Lactanet, Sainte-Anne-de-Bellevue, QC, Canada) for analysis of fat, protein, true protein, lactose, total solids, milk urea nitrogen (MUN) and SCC.

### 2.5. Enteric Methane Production

The respiration chambers measured 4.09 m long × 2.95 m wide × 2.84 m high. When chamber doors were closed, air entered the chamber through a ventilation duct and exited through an exhaust duct. Air temperature inside the chambers was maintained at 16.8 ± 0.16 °C. Air flow into and out of the chambers was measured using inline mass flowmeters (FT2; Fox Thermal Instruments Inc., Marina, CA, USA) and was maintained at 180 ± 10 m^3^/h. Methane concentration was continuously measured at the air entrance and exhaust ducts using CH_4_ analyzers (SERVOPRO 4100; Servomex, Brighton, East Sussex, UK). The amount of CH_4_ (entering and leaving the chamber) was calculated by multiplying the concentration of CH_4_ by the airflow (at entrance and exhaust ducts). The difference between the incoming and outgoing mass of CH_4_ corresponded to the amount of enteric CH_4_ emitted in each chamber by the animal. The chambers were calibrated at the beginning of each experimental period by releasing known amounts of CH_4_ in each chamber (with no cow inside). The calibration factors (i.e., to adjust each chamber to 100% recovery) specific to each experimental period were used to correct CH_4_ emissions data. A small positive pressure was generated inside each chamber to prevent inflow of gases into the chambers. Methane was recorded every minute over 5 consecutive days and fluxes were averaged to derive 24-h CH_4_ emissions. Cows were preconditioned to the environmental chambers before the beginning of the experiment. To reduce the effect of isolation on animal behavior, the chambers were equipped with windows and speakers so the cow in the chamber could see and hear other cows. Cows entered the chambers 18 h before starting CH_4_ measurements. Within each chamber, the cow was kept in a tie stall measuring 1.82 m long × 1.60 m wide that was elevated 25 cm above the floor. Manure was collected from each cow in a stainless box placed below and to the rear of each stall. To feed and milk the cows and remove the manure, farm personnel accessed the chambers twice daily and the doors were kept opened for 1 to 1.5 min to allow exchange of material in and out of the chambers. This resulted in interruptions of flux measurements for 15 to 30 min, which is the time required for gas concentrations to reach steady state. Missing concentrations of CH_4_ during these interruptions were estimated as the average of the CH_4_ concentration immediately before the interruption and immediately after gas concentration reached steady state. These interruptions had little effect on daily emissions because fluxes were calculated every minute and used to derive the 24-h period emissions values. Cows in the chambers were milked twice daily, had free access to water, and were fed for ad libitum intake (5% orts on an as-fed basis). Offered feed and orts were weighed daily to determine feed consumption. Samples of offered feed and orts were collected, pooled across days, and kept frozen for later determination of DM and GE concentrations.

### 2.6. Chemical Analyses

Dry matter content was determined by drying samples in a vacuum oven at 100 °C overnight (method 934.01; [29]). Ash content was determined by sample incineration at 550 °C overnight in a muffle furnace (method 942.05; [29]). Organic matter content was calculated as the difference between 100 and the percentage of ash. Crude protein (N × 6.25) was determined using a LECO N analyzer (TruMac N determinator, LECO Corporation, St. Joseph, MI, USA) according to AOAC [29] (method 990.03). The concentration of NDF was determined as described by Van Soest et al. [30] with the use of sodium sulfite and the inclusion of heat stable α-amylase. The ADF content was determined according to AOAC [29] (method 973.18). The procedures for NDF and ADF were adapted for use in an Ankom 200 Fiber Analyzer (Ankom Technology Corp., Fairport, NY, USA). The concentration of starch was determined colorimetrically according to AOAC [29] (method 996.11). Energy concentration in diet, feces, urine, and milk samples was determined using an oxygen bomb calorimeter (model 6200, Parr Instrument Company, Moline, IL, USA). The concentration of N in acidified urine samples was determined by micro-Kjeldahl analysis [29]. Purine derivatives (allantoin and uric acid) in urine were analyzed according to the procedure of George et al. [31] using HPLC (model 210, Varian ProStar, Agilent Technologies Canada Inc., Montreal, QC, Canada). Analysis of VFA was performed using a gas chromatography (GC) equipped with a flame ionization detector and auto-injector (6850 network GC system, Agilent Technologies, Mississauga, ON, Canada) fitted with a DB-FFAP column (30 m × 0.250 mm × 0.25 µm; Agilent Technologies, Mississauga, ON, Canada). Ammonia concentration was determined as in Weatherburn [32]. Total protein, true protein, fat, lactose, total solids, MUN, and SCC in milk samples were analyzed by infrared spectroscopy (MilkoScan FT 6000; Foss Electric, Hillerød, Denmark). Milk composition was adjusted taking into account differences in milk yield between a.m. and p.m. milkings.

### 2.7. Statistical Analyses

Data were analyzed using the MIXED procedure of SAS (SAS Institute Inc., Cary, NC, USA) and the statistical model included breed, MP supply, interaction of breed × MP supply, and period as fixed effects and cow within breed as random effect. Concentrations of VFA and NH_3_, and protozoa density data, were analyzed as repeated measures using the same model with the addition of the fixed effects of day, sampling time (i.e., hour) and all interactions. The CH_4_ data were also analyzed as repeated measures with the inclusion of the fixed effects of day and interaction of day with study factors. The appropriate covariance structure used for repeated-measures analyses was chosen according to the type of repeated measurement (single or double), sampling interval (equal or unequal) and to achieve the lowest Akaike, corrected Akaike, and Bayesian information criteria values. Therefore, the choice of covariance structure varied with the response variable. The covariance structure used cow within treatment × period as subject of the repeated measures. Orthogonal polynomial contrasts (linear and quadratic) were used to examine MP supply effect on response variables. Differences between treatments (i.e., main effects: breed and MP supply) and their interactions (i.e., Breed × MP supply) were declared significant at *p* ≤ 0.05 and tendencies for 0.05 < *p* ≤ 0.10. Data are reported as least square means with standard error of the means (SEM).

## 3. Results

### 3.1. Milk Production and Milk Composition

Data of DMI, milk production, milk composition, and feed or N efficiency are presented in Table 2. The interaction of breed × dietary MP supply was significant (*p* = 0.05) only for milk yield. The milk yield of Ayrshire cows increased linearly as the dietary MP level increased whereas a quadratic increase was observed for Holstein cows (i.e., no further increase from 100% to 115% MP supply).

Overall, Ayrshire cows produced 25% less (*p* < 0.01) milk than Holstein cows (32.8 vs. 43.7 kg/d). Concentrations of fat, protein, and total solids were 0.3, 0.3, and 0.5 percentage units greater (*p* ≤ 0.06) in the milk of Ayrshire cows than in the milk of Holstein cows. Milk lactose concentration averaged 4.66% and was not affected by breed. Ayrshire cows produced (kg/d) less (*p* < 0.01) milk fat (−19%) and milk protein (−16%) than Holstein cows. Consequently, yield ECM was less (*p* < 0.01) for Ayrshire cows compared with Holstein cows (36.0 kg/d vs. 45.3 kg/d, respectively). Concentrations of MUN tended to be less (*p* = 0.07) for Ayrshire cows compared with Holstein cows (8.78 vs. 9.90 mg/dL). The intake of DM was 20% less for Ayrshire cows than for Holstein cows, but did not differ between the two breeds when expressed as a percentage of BW (average 3.66%). Feed efficiency expressed as kg of milk/kg of DMI tended (*p* = 0.10) to be less for Ayrshire cows compared with Holstein cows (1.60 vs. 1.69 g milk/kg DMI), but did not differ between the two breeds, if expressed on an ECM yield basis. Likewise, N use efficiency (g N in milk/100 g of N intake) was not different between Ayrshire cows and Holstein cows.

Milk protein concentration increased (*p* = 0.02) with increasing MP supply from 85% to 100% but no further increase was observed when MP supply increased from 100% to 115% (i.e., quadratic response). However, concentrations of fat, lactose, and total solids were not affected by MP supply. Consequently, the yield (kg/d) of milk protein increased (*p* ≤ 0.01) in a quadratic manner (i.e., no additional increase from 100% to 115% MP supply. The yields of fat and lactose increased linearly (*p* < 0.01) with increasing MP supply, with a quadratic tendency for milk lactose (*p* = 0.10). The yield of ECM increased linearly (*p* < 0.01) as MP supply increased, but with a quadratic tendency (*p* = 0.07). Increasing MP supply was associated with a numerical increase (*p* = 0.12) in DMI (kg/d) but had no effect on DMI expressed on a BW basis. A linear increase (*p* < 0.01) was observed for feed efficiency (kg ECM/kg DMI) as dietary MP supply increased. However, N use efficiency decreased linearly (*p* < 0.01) with increasing MP supply.

### 3.2. Ruminal Fermentation Characteristics

Data of continuous measurements of ruminal pH are presented in Table 3. A tendency (*p* = 0.07) for breed × MP supply interaction was observed for the mean daily ruminal pH: pH declined linearly in Ayrshire cows as MP supply increased but was not affected in Holstein cows. Regardless of breed and dietary MP supply, the minimum and maximum ruminal pH averaged 5.78 and 6.81, respectively. Daily time spent at pH < 6.0 averaged 205 min/d whereas time spent at pH < 5.6 was negligible and averaged 8 min/d.

Because interactions between breed, dietary MP supply, measurement day, and sampling time were not significant for VFA, NH_3_, and protozoa, only averages of the 2 d measurements and the sampling times are reported (Table 4). No effects of breed or MP supply were observed for total VFA concentration, the molar proportions of individual VFA, the acetate:propionate ratio, or NH_3_ concentration. Protozoa density was less in Ayrshire cows than in Holstein cows (4.4 × 105 vs. 6.3 × 105/mL) and was unaffected by dietary MP supply.

### 3.3. Apparent Total-Tract Digestibility

The effects of breed, dietary MP supply, and their interaction on apparent total-tract digestibility are presented in Table 5. The apparent total-tract digestibility of DM, OM, crude protein (CP), NDF, and GE was greater (*p* ≤ 0.05) in Ayrshire cows than in Holstein cows. Starch digestibility averaged 98% and was not affected by breed. A tendency for a linear increase (0.5 < *p* ≤ 0.10) in the digestibility of OM and GE was observed as dietary MP supply increased, mainly related to increased (*p* ≤ 0.04) CP and starch digestibility. However, the apparent total-tract digestibility of DM, NDF, or ADF was not affected by dietary MP supply.

### 3.4. Methane Production

Interactions between breed, dietary MP level, and measurement days were not significant for CH_4_ data, and therefore, only averages across measurement days are presented in Table 6. Similar to the response observed during the 7-d collection period, DMI was 22% less (*p* < 0.01) for Ayrshire cows than for Holstein cows and increased linearly (*p* = 0.01) with increasing MP supply. Daily CH_4_ emissions were 20% less (*p* = 0.06) for Ayrshire cows compared with Holstein cows (386 vs. 484 g/d) and increased linearly (*p* = 0.03) as the MP supply increased. However, there was no effect of breed or MP supply on CH_4_ yield (averaging 18.9 g/kg DMI) or CH_4_ intensity (averaging 10.8 g/kg ECM).

### 3.5. Energy Utilization

Data of energy intake, expenditure, and partitioning are presented in Table 7. Compared with Holsteins cows, the intakes of gross, digestible, metabolizable, or net energy were less (by 21, 18, 18, and 18%, respectively; *p* = 0.01) for Ayrshire cows than for Holstein cows. There was no effect of dietary MP supply on energy intake variables. Daily fecal, urinary, CH_4_, and milk energy expenditures (Mcal/d) were less (*p* ≤ 0.06) for Ayrshire cows than for Holstein cows (−26%, −18%, −20%, and −21%, respectively). However, when expressed as a percentage of GE intake, energy expenditures in urine, CH_4_, and milk were not affected by breed. Only fecal energy loss was less in Ayrshire cows than in Holstein cows (30.0% vs. 32.3%, *p* = 0.02).

Increasing the dietary MP supply linearly (*p* ≤ 0.03) increased the daily energy expenditure in urine, CH_4_, and milk, but had no effect on the daily energy expenditure in feces. When expressed as a percentage of GE intake, urine and milk energy expenditures increased linearly (*p* ≤ 0.04) when the dietary MP supply increased. Energy expenditure in CH_4_ was unaffected whereas fecal energy expenditure tended (*p* = 0.09) to linearly decrease with increasing MP supply. The maintenance energy expressed as a percentage of GE intake was not affected by breed or MP supply. The proportion of metabolizable energy (ME) used for milk energy averaged 42.7% and was unaffected by breed, but tended (*p* = 0.10) to increase linearly with increasing MP supply.

### 3.6. Nitrogen Utilization

Data of N intake and excretion are presented in Table 8. The less N intake for Ayrshire cows resulted in reduced (*p* ≤ 0.02) daily N excretion (feces, urine, and total) compared with Holstein cows. When expressed as a percentage of N intake, urinary N excretion was not affected by breed. In contrast, fecal N and total N excretions relative to N intake were less (*p* = 0.01) for Ayrshire cows than for Holstein cows. The daily urine excretion of allantoin and uric acid was less (*p* ≤ 0.01) in Ayrshire than Holstein cows.

The daily N intake and total (urine, total) N excretion increased linearly (*p* < 0.01) whereas fecal N excretion tended (*p* = 0.08) to increase as the MP supply increased. The increase in the amount of N daily excreted in urine was of a greater magnitude for Holstein cows than for Ayrshire cows, explaining the tendency (*p* = 0.07) of the interaction of breed × MP supply. When expressed as a percentage of N intake, fecal N excretion decreased linearly (*p* < 0.01) whereas urinary N and total N excretion increased linearly (*p* ≤ 0.01) with increasing MP supply. The daily urine excretion of purine derivatives (mmol/d) were not affected by an increasing MP supply.

## 4. Discussion

Different nutritional models are available for dairy cow diet formulation. In our study, the CNCPS model (version 6.5/6.55) was used. This model is widely used to formulate diets of dairy cattle with an updated feed library [35] and the version used (6.5/6.55) integrated the most recent findings [36]. Nutrient (e.g., metabolizable MP) requirements are model dependent and the use of other models (e.g., NRC [14]) may result in different predictions than those from the CNCPS model. Nevertheless, these predictions (i.e., MP supply) are more accurate than CP in estimating the AA availability to the cows.

Diets are formulated to meet the nutrient requirements of the dairy cow, particularly energy (ME) and protein (MP) requirements. Such requirements vary according to DMI, milk yield, milk component, BW, stage of lactation (DIM), parity, etc. The goal is to supply adequate amounts of nutrients allowing the cows to express their full genetic potential of production. Because Holstein and Ayrshire cows are breeds that differ in their physical characteristics (e.g., BW, rumen size, potential DMI) and their genetic potential of milk production (i.e., yield and composition), meeting nutrient requirements for milk production requires formulating a diet (e.g., 100% MP) specific to each breed. If fed the same experimental diets as Holstein cows, the Ayrshire cows would have been underfed relative to their requirements and would be in a more responsive “status”, which would have totally confounded the interpretation of the results. For instance, the diets formulated to supply 85%, 100%, and 115% of the MP requirement of Holstein cows would meet only 81%, 94%, and 109% of the MP requirement of Ayrshire cows (Table 9), respectively. Thus, feeding diets specific to each breed versus feeding the same diet to both breeds was appropriate to evaluate each breed responses to increasing MP supply (85%, 100%, and 115%).

### 4.1. Breed × Dietary MP Level Interaction

With the exception of milk yield, the two breeds responded similarly to changes in dietary MP supply as shown by the absence of breed × dietary MP supply interaction for almost all the measured variables (see discussion below). In Ayrshire cows, milk yield increased linearly with increasing MP supply whereas a quadratic response was observed in Holstein cows (i.e., no additional increase from 100% to 115% MP supply). This interaction indicates that the MP requirements of the Ayrshire breed for milk production may be greater than those predicted by CNCPS. However, the breed × MP supply interaction was not significant for ECM or milk component yields (*p* ≥ 0.16). To our knowledge, this is the first study investigating the responses of Ayrshire vs. Holstein cows to variations in MP supply.

### 4.2. Effect of Breed

From DHI reports in Canada [3,4,5], Ayrshire cows are typically 15% lighter than Holstein cows and produce 25, 23, and 23% less milk, milk fat, and milk protein, respectively, than Holstein cows. These national averages are similar to the differences between breeds observed in the present study, except for milk protein yield (16% less in Ayrshire cows vs. Holstein cows in this study). From DHI data and bulk milk analyses in Pennsylvania, Cerbulis and Farrell [37] reported that Ayrshire cows produced milk with 7% and 10% higher concentrations of milk protein and fat, but only 5% less milk yield than Holstein cows, yielding a 6% greater milk fat yield and a similar milk protein yield. On the other hand, in a quantitative genetic study on cows kept in different sites, McAllister et al. [38] reported that Ayrshire cows produce 29, 31, and 24% less milk, milk protein, and milk fat than Holstein cows. According to Lactanet [3,4,5], concentrations of fat and protein are greater (0.13 and 0.12 more percentage units) in the milk of Ayrshire than in that of Holstein cows. In the present study, where the two breeds were under controlled-experimental conditions, milk fat and milk protein concentrations were also greater for Ayrshire cows than for Holstein cows (0.33 and 0.33 percentage units more, respectively). The evolution of the two breeds throughout the past decades necessitates revising the energy and protein requirements of Ayrshire cows as more attention has been given to defining the nutrient requirements of Holstein cows. Differences in MP requirement between different dairy cattle breeds have been rarely addressed.

In lactating dairy cows, intake is primarily driven by ECM yield, followed by BW [14,39,40]. When expressed on a BW basis, DMI observed in this study averaged 3.66% (18.3 kg/100 kg metabolic BW, data not shown) and was not affected by breed, suggesting that BW was the main driving factor of DMI. The less DMI (20% lower) observed in Ayrshire cows was mainly due to the lower ECM yield (21% lower) which resulted in no change in feed efficiency (kg ECM/kg DMI) between the two breeds in response to an increasing MP supply.

The efficiency of energy use (Mcal milk energy/100 Mcal GEI; Mcal milk energy/100 Mcal ME intake) or N use (g milk N/100 g N intake) were not different between the two breeds despite lower GE, ME, and N intakes (−21%, −18%, and −15%, respectively) in Ayrshire cows compared with Holstein. The greater DM, GE, and CP apparent total-tract digestibility in Ayrshire cows versus Holstein cows are more likely due to the lower forage percentage (4 percentage units) in Ayrshire diets compared with Holstein diets. Alternatively, Ayrshire cows (smaller breed than Holstein) may have a bigger gastro-intestinal tract relative to BW. To our knowledge, there are no reports that compare gastro-intestinal tract size between Ayrshire and Holstein cows. When adjusted to BW, Beecher et al. [10] reported that Jersey cows (smaller than Holstein cows) have a bigger gastro-intestinal tract (143 g/kg BW) than Holstein cows (129 g/kg BW). In this study, at similar potential DMI (as a percentage of BW) and the potentially larger gastro-intestinal tract in Ayrshire cows, the ruminal passage rate may have been slower in Ayrshire cows than in Holstein cows, which may have increased fiber (and consequently OM) digestibility, compared with Holstein cows. An increase in fiber retention time in the rumen (i.e., slower passage rate) is expected to enhance fiber digestibility [41], which was reflected in the current study by the greater fiber (NDF, ADF) digestibility in Ayrshire cows compared with Holstein cows. When Jersey and Holstein cows were fed similar diets, Aikman et al. [42] and Olijhoek et al. [11] reported higher NDF digestibility in Jersey cows. However, this difference did not translate into improved feed efficiency; energy losses (relative to GEI) in urine, CH_4_, and N loss in urine were not different between the two breeds.

Studies investigating energy and N use efficiencies between breeds are scarce. In agreement with our findings, Dickinson et al. [12] reported no difference in energy efficiency (milk energy/GEI) between Ayrshire and Holstein first parity cows. Likewise, Dong et al. [13] reported no effect of dairy breed (Holstein; Jersey × Holstein; Norwegian × Holstein) on energy partitioning. Kristensen et al. [43] observed no difference in the efficiency of N use between Holstein, Jersey, or other breeds of dairy cattle. Therefore, data from this study and others suggest that Ayrshire cows do not have an advantage over Holstein cows in energy and N use efficiency for milk production.

The difference in DMI between the two breeds (5.8 kg/d less for Ayrshire cows) accounts for the fewer (98 g/d) daily enteric CH_4_ emissions from Ayrshire cows compared with Holstein cows. In contrast, CH_4_ yield (18.9 g/kg DMI) and intensity (10.8 g/kg ECM) were not affected by breed. The yield and intensity of the CH_4_ emissions of Ayrshire cows (19.0 g/kg DMI and 12.1 g/kg milk, respectively) are close to the mean values based on a large number of observations for Holstein cows [44]. Other studies reported no differences in CH_4_ yield and intensity between different breeds of dairy cattle [45,46], beef cattle [47,48], or even between dairy and beef cattle breeds [49] fed similar diets. The absence of variation in ruminal methanogen populations between breeds may explain such similar enteric CH_4_ yields. King et al. [50] reported 85% similarity in the methanogen population between Holstein and Jersey breeds. Others also reported similarity in methanogen communities between dairy cattle breeds [51], beef cattle breeds [48], or between dairy and beef cattle [49].

The absence of breed effect on ruminal VFA (total concentration and molar proportions) is in line with the lack of breed effect on CH_4_ yield. In accordance, others reported no effect of breed of dairy cattle [52] or beef cattle [48] on ruminal VFA concentration or pattern. The lower density of protozoa in the ruminal content of Ayrshire cows compared with that of Holstein cows was probably due to less DMI in Ayrshire cows as a positive relationship between DMI and the protozoa population in the rumen, as has been previously reported [53,54,55]. In crossbred Charolais and purebred Luing (LU) beef steers with similar DMI, breed had no effect on ruminal protozoa population [48].

### 4.3. Effect of MP Supply

The CNCPS model was used to formulate diets to meet the MP requirements (100%) of Ayrshire cows and Holstein cows, based on BW, milk production and milk composition, and DIM. When MP supply was reduced to 85% or increased to 115% of the requirement, RDP was kept adequate (≥9.5% DM) to maintain ruminal health [56,57,58]. Reducing MP supply to 85% involved decreasing the proportions of ingredients with elevated RUP content (i.e., soybean meal, heat-treated soybean meal, corn gluten meal). In contrast, increasing MP supply to 115% was achieved by increasing dietary proportions of heat-treated soybean meal and corn gluten meal. As a consequence, dietary CP concentration increased with MP supply.

The quadratic increase in ECM yield, and the yield and concentration of milk protein with an increased dietary MP supply resulted from the large increase when MP supply increased from 85% to 100%, but a small (or no) increase when MP supply increased further to 115%. Meanwhile, increasing MP supply caused a linear increase in milk fat and milk lactose yields, with quadratic tendencies for milk lactose yield. The observed increase in performance was not associated with increased gross, digestible, metabolizable, or net energy intakes and is therefore solely related to the increased MP supply. Rather, this increase was related to the increase in CP intake and digestibility as dietary MP level increased.

The lack of MP supply effect on ruminal NH_3_ concentration and urinary allantoin and uric acid suggest that microbial protein synthesis was unaffected, probably because of the adequate dietary RDP supply. Therefore, the increase in RUP contribution to MP supply is the main reason for the enhanced performance. In agreement, Broderick [18] reported an increase in milk, fat, and protein yields when dietary RUP (% of DM) increased from 4.83% (CP = 15.1% of DM) to 5.48% (CP = 16.6% of DM). However, no further increases were observed when RUP increased to 6.22% (CP = 18.3% of DM) in that study. Bahrami-Yekdangi et al. [59] reported a quadratic response of milk production to increasing MP supply with RUP increasing from 4.7% to 7.1% of DM: the lowest and the highest milk yield occurred at 4.7% RUP (CP = 15.1% of DM) and 5.5% RUP (CP = 16.4% of DM), respectively, but no response was observed when RUP increased to 6.3% and 7.1% (CP = 17.2% and 18.0% of DM, respectively). A meta-analysis by Huhtanen and Hristov [19], using a large database from North America and Europe, revealed a quadratic response of milk protein yield and N use efficiency to increased MP supply, with a diminishing response when MP supply is above MP requirement. It is worth mentioning that the studies of Broderick [18] and Bahrami-Yekdangi et al. [59] used the NRC model to predict MP supply to meet the protein requirement of the cows, whereas the current study used the CNCPS model. Therefore, predictions from various studies can be only compared with caution because of the inevitable differences between the models.

In the present study, feeding the 85% MP diets (RUP = 4.24% and 3.64% for Ayrshire cows and Holstein cows, respectively) was not sufficient to support maximal yields of milk and milk components, especially milk protein. Lee et al. [60] reported that at similar DMI, cows produced 3.1 kg/d less milk when fed a RUP-deficient diet (RUP = 4.9% of DM, CP = 14.8%) compared with a diet that met RUP requirements (RUP = 6.1% of DM, CP = 17.6%). In another study, Metcalf et al. [61] showed that reducing RUP (and consequently MP) from above (+12.5%) to below (−12.5%) the requirement reduced milk production by 2.3 kg/d without impairing DMI. Blouin et al. [62] reported that reducing dietary RUP concentration while maintaining an adequate RDP level caused a decrease in the net portal absorption of total and essential AA and consequently lowered milk and milk protein yields. In the present study, the intestinal availability of AA may have been reduced when supplying 85% versus 100% MP. Such changes may be related to decreases in intake and the digestion of CP and to the lesser availability of AA from RUP when supplying 85% MP versus 100% MP.

The increase in apparent CP digestibility with increasing MP supply was probably due to: (1) more CP intake associated with highly digestible CP sources (i.e., soybean and corn gluten meals) when supplying 115 versus 85% MP, and (2) a reduced proportion of metabolic endogenous fecal N at a greater MP supply relative to total fecal N compared with a relatively greater contribution at a lower MP supply. Broderick [18] and Lee et al. [60] also reported an increase in CP digestibility when RUP content increased. Based on these considerations, in both breeds, providing 85% of the MP requirement (versus 100% MP) may have limited AA availability for milk protein synthesis and consequently decreased milk production.

In the current study, supplying 85% of the MP requirement impaired feed efficiency, expressed as kg of ECM/kg of DMI. In agreement, data of Metcalf et al. [61] indicate a depressed feed efficiency when MP supply requirement was reduced by 12.5 and 25% mainly through reducing RUP sources in the diet. In contrast, using the NRC model to predict MP requirement, Broderick [18], Lee et al. [60], and Bahrami-Yekdangi et al. [59] reported no effect of MP supply on feed efficiency in Holstein cows. Those studies identified diets as low or deficient when RUP concentration was 4.1–4.8% of DM, which is greater than the RUP concentration (3.6% of DM) corresponding to the 85% MP supply in the present study. On the other hand, N use efficiency were increased at 85% MP supply. Metcalf et al. [61] and Laroche et al. [63] both reported the improved efficiency of MP conversion to milk protein when the MP supply was 10 or 25% below the animal requirement.

Fecal and urinary N excretion and energy losses in urine decreased when the MP supply was limited to 85% of the animal requirement. This was mainly due to the reduced (−14%) N intake accompanied with a smaller (−5%) decrease in milk N output when the MP supply was limited to 85% versus 100% of the animal needs. At 85% MP supply, the decrease in urinary N excretion is consistent with the reduced MUN concentration. However, such a decrease cannot be related to changes in ruminal N metabolism because ruminal NH_3_ concentration was unaffected, more likely due to the similar dietary RDP concentration among the experimental diets. Thus, the possible reduction in AA catabolism resulting from a reduced supply and the consequent potential decrease in blood AA concentrations may explain the observed decreases in MUN concentration and urinary N excretion. Others [18,59,60] reported that reducing dietary RUP concentration or MP supply did not affect ruminal NH_3_ concentration, but reduced MUN and urinary N excretion.

No additional increase in ECM or milk protein yields was achieved when MP supply increased from 100 to 115% (i.e., 438 and 385 g/d of RUP). This lack of response suggests that, at 100% MP supply, protein requirements were fulfilled and providing over MP requirement does not result in an additional gain in ECM or milk protein yields. In agreement, McCormick et al. [64], Broderick [18], and Bahrami-Yekdangi et al. [59] reported no further increase in FCM, ECM, or milk protein yields when MP supply (and RUP) increased above the requirement of early- and mid-lactation Holstein cows.

In the present study, as a result of the linear increase in DMI as MP supply increases during the measurement of CH_4_ emissions, daily enteric CH_4_ emission (g/d) also increased given the well-established positive relationship between DMI and the amount of enteric CH_4_ emitted [65,66,67]. The yield (g/kg of DMI, %GE intake) and emission intensity (g/kg of ECM) of CH_4_ were unaffected by the experimental treatments because of the changes that occurred in the same directions in the nominators and denominators of these ratios. Van Dorland et al. [68] observed an increase in enteric CH_4_ emissions associated with a numerical increase in DMI but no effect on milk yield in cows fed a ryegrass-based diet supplemented with corn gluten meal (i.e., RUP source). On the other hand, Hynes et al. [69,70] and Niu et al. [71] reported no change in DMI, milk yield, or daily enteric CH_4_ production in dairy cows fed diets that were supplemented with RUP sources (soybean meal and/or canola meal). As a consequence, CH_4_ emission yield and intensity were unaffected in the studies of van Dorland et al. [68], Hynes et al. [69], and Niu et al. [71].

## 5. Conclusions

In this study, the CNCPS (version 6.55) was used to formulate diets supplying 85%, 100%, or 115% of the MP requirement to Ayrshire and Holstein cows. Dry matter intake and milk production were less for Ayrshire cows than for Holstein cows. However, feed efficiency (kg of ECM/kg of DMI), efficiencies of the utilization of energy (milk Mcal/100 ME intake) and dietary protein for milk protein synthesis (g N milk/100 g N intake), and urinary N (g/100 g N intake) did not differ between the two breeds. Despite the greater CH_4_ emissions (g/d) by Holstein cows versus Ayrshire cows (as a result of a greater DMI), CH_4_ yield (g/kg DMI) was not different between the two breeds. Methane intensity (g/kg ECM) was also unaffected by breed, despite Ayrshire cows producing less milk than Holstein cows. Results from this study show that the responses of Ayrshire cows to an increased MP supply were not different from those of Holstein cows as energy-corrected milk or milk protein yields were not affected by breed × MP supply interaction, whereas urinary N excretion increased as dietary MP supply increased.

## Figures and Tables

**Table 1:** Ingredient and chemical composition of the experimental diets formulated to provide 85%, 100%, or 115% of MP requirement.

	Holstein Diets			Ayrshire Diets		
	MP			MP		
Item (% of DM, Unless Otherwise Noted)	85%	100%	115%	85%	100%	115%
Ingredient						
Corn silage	45.9	45.6	45.6	39.3	39.3	39.6
Alfalfa silage	17.3	17.2	17.5	18.5	18.6	19.1
Corn grain, ground	20.1	16.1	15.1	21.2	19.0	15.4
Soybean meal, 48% solvent-extracted	4.06	7.84	1.53	7.19	8.29	4.14
Soybean meal, heat-treated ^1^	0	2.10	8.16	0	1.65	7.84
Soybean hulls	3.18	1.40	-	3.59	1.04	0
Timothy hay, chopped	3.56	3.54	3.48	4.16	4.17	4.17
Corn gluten meal	0	0.78	3.11	0	2.61	4.27
Rumen inert fat supplement ^2^	2.70	2.25	2.44	2.87	2.21	2.65
Urea	0.50	0.28	0.28	0.38	0.10	0
Mineral and vitamin supplement ^3^	1.48	1.47	1.47	1.56	1.56	1.56
Calcium carbonate	0.57	0.68	0.65	0.45	0.63	0.54
Sodium bicarbonate	0.69	0.69	0.69	0.81	0.81	0.81
Chemical composition						
DM	43.1	43.0	42.7	43.2	43.2	42.8
OM	93.0	93.0	92.8	93.3	92.9	93.1
CP	13.1	15.4	16.5	14.3	16.1	17.5
Soluble N, % total N	52.7	40.5	36.9	43.5	33.8	28.5
NPN, % total N	40.8	30.7	28.1	34.4	25.2	21.3
NDIN, % total N	10.9	11.2	13.5	10.8	11.4	13.9
ADIN, % total N	3.80	3.63	4.20	3.73	4.11	4.40
RUP, % DM ^4^	3.64	5.11	6.81	4.24	5.90	7.66
RDP, % DM ^4^	9.50	10.26	9.73	10.03	10.17	9.88
MP, % DM ^4^	8.97	10.42	11.95	9.66	11.25	12.89
NDF	28.4	26.9	27.4	28.2	27.9	27.7
ADF	18.9	17.5	18.2	18.4	18.5	18.3
Starch	30.7	29.9	27.6	31.8	28.8	28.4
Ether extract	4.62	4.15	4.54	4.76	4.33	4.52
Gross energy, Mcal/kg DM	4.52	4.53	4.58	4.50	4.52	4.55
Metabolizable energy, Mcal/kg DM ^4^	2.66	2.67	2.69	2.65	2.65	2.67

^1^ Rupro Soy^®^ (Benjamin Milles, St-Césaire, QC, Canada) ^2^ Energy Booster^®^ 100 (Milk Specialties Global Animal Nutrition, Carpentersville, IL, USA). ^3^ Contained 12.48% Ca, 6.80% P, 6.81% S, 7.72% Na, 1.97% K, 96 mg/kg I, 2877 mg/kg Fe, 620 mg/kg Cu, 2520 mg/kg Mn, 3777 mg/kg Zn, 83 mg/kg Co, 628,000 IU/kg vitamin A, 81,000 IU/kg vitamin D, 3739 IU/kg vitamin E, and 27.8 mg/kg Se. ^4^ Estimated using CNCPS (version 6.55) at DMI of 21.6 and 26.0 kg/d for Ayrshire and Holstein cows, respectively.

**Table 2:** Dry matter intake, milk production, and milk composition of lactating Holstein and Ayrshire cows fed diets formulated to provide 85%, 100%, or 115% of metabolizable protein (MP) requirement ^1^.

	Holstein				Ayrshire							
	MP			SEM	MP			SEM	*p*			
	85%	100%	115%		85%	100%	115%		Breed	MP		Breed × MP
Item										Linear	Quadratic	
DMI, kg/d	25.4	26.0	25.9	0.81	20.2	20.6	20.8	0.79	<0.01	0.12	0.44	0.84
DMI, % BW	3.65	3.74	3.71	0.143	3.58	3.64	3.65	0.135	0.70	0.20	0.32	0.88
BW, kg	700	699	701	18.7	570	569	574	18.7	<0.01	0.39	0.37	0.76
MP intake, g/d	2278	2715	3092	93.1	1956	2317	2686	106.2	0.01	<0.01	0.82	0.75
Production, kg/d												
Milk	41.9	45.0	44.3	1.77	31.7	32.7	34.1	1.67	<0.01	<0.01	0.04	0.05
4% FCM ^2^	41.0	43.3	42.9	1.64	31.9	33.1	34.4	1.61	<0.01	<0.01	0.15	0.30
ECM ^3^	43.6	46.5	45.9	1.71	34.5	36.1	37.4	1.66	<0.01	<0.01	0.07	0.28
Composition												
Fat, %	3.79	3.68	3.73	0.128	4.03	4.08	4.08	0.128	0.06	0.90	0.70	0.49
CP, %	2.99	3.09	3.08	0.062	3.33	3.44	3.39	0.059	<0.01	0.03	0.02	0.80
True protein, %	2.80	2.90	2.88	0.061	3.14	3.25	3.20	0.060	<0.01	0.03	0.02	0.82
Lactose, %	4.72	4.66	4.67	0.041	4.63	4.64	4.62	0.032	0.21	0.22	0.54	0.42
Total solids, %	12.5	12.4	12.5	0.18	12.9	13.1	13.0	0.18	0.03	0.67	0.64	0.38
SCC, log_10_/mL	4.37	4.42	4.40	0.156	4.35	4.43	4.37	0.136	0.94	0.66	0.35	0.93
Yield, kg/d												
Fat	1.61	1.68	1.68	0.074	1.28	1.34	1.38	0.071	<0.01	<0.01	0.37	0.61
Protein	1.25	1.38	1.35	0.050	1.06	1.13	1.15	0.046	<0.01	<0.01	0.01	0.31
Lactose	1.97	2.10	2.07	0.074	1.47	1.52	1.57	0.073	<0.01	<0.01	0.10	0.16
MUN, mg/dL	6.67	10.41	12.62	0.537	6.07	8.88	11.4	0.494	0.07	<0.01	0.18	0.47
Feed efficiency												
Milk/DMI	1.65	1.72	1.71	0.043	1.57	1.59	1.64	0.042	0.10	<0.01	0.36	0.36
ECM/DMI	1.71	1.78	1.77	0.030	1.71	1.75	1.80	0.032	0.97	<0.01	0.59	0.58
N use efficiency, g N milk/100 g N intake	36.8	33.9	31.1	0.78	35.9	33.3	30.9	0.59	0.53	<0.01	0.84	0.71

^1^ Least square means from 6 Latin squares (18 cows) ^2^ 4% fat corrected milk (FCM) [33] = 0.4 × milk yield (kg/d) + 15 × fat yield (kg/d). ^3^ ECM [33] = 0.327 × milk yield (kg/d) + 12.95 × fat yield (kg/d) + 7.2 × protein yield (kg/d).

**Table 3:** Ruminal pH of lactating Holstein and Ayrshire cows fed diets formulated to provide 85%, 100%, or 115% of metabolizable protein (MP) requirement ^1^.

	Holstein				Ayrshire							
	MP			SEM	MP			SEM	*p*			
	85%	100%	115%		85%	100%	115%		Breed	MP		Breed × MP
Item										Linear	Quadratic	
pH												
Mean	6.29	6.32	6.31	0.086	6.44	6.29	6.27	0.083	0.82	0.10	0.53	0.07
Minimum	5.74	5.72	5.77	0.105	5.92	5.75	5.75	0.130	0.67	0.23	0.20	0.21
Maximum	6.74	6.84	6.78	0.061	6.88	6.76	6.83	0.060	0.59	0.89	0.84	0.11
Time pH < 6.0, min/d	223	154	133	140.0	140	280	298	132.9	0.67	0.71	0.81	0.37
Time pH < 5.6, min/d	20	7	1	8.3	0	14	4	6.6	0.60	0.29	0.52	0.13

^1^ Least square means from 4 Latin squares (12 cows).

**Table 4:** Total VFA concentration, VFA molar proportions, NH_3_ concentration, and protozoa number in ruminal fluid of lactating Holstein and Ayrshire cows fed diets formulated to provide 85%, 100%, or 115% of metabolizable protein (MP) requirement ^1^.

	Holstein				Ayrshire							
	MP			SEM	MP			SEM	*p*			
	85%	100%	115%		85%	100%	115%		Breed	MP		Breed × MP
Item										Linear	Quadratic	
Total VFA, mM	134	134	137	4.1	131	135	131	4.2	0.60	0.59	0.67	0.46
VFA, mol/100 mol												
Acetate	63.9	62.5	63.3	1.48	61.5	61.0	60.5	1.57	0.26	0.37	0.45	0.73
Propionate	19.1	20.1	20.2	1.83	22.5	23.4	22.9	1.82	0.17	0.45	0.53	0.91
Butyrate	11.1	11.7	10.9	0.38	10.8	10.5	11.6	0.57	0.61	0.46	0.98	0.11
Isobutyrate	0.88	0.87	0.83	0.315	0.89	0.88	0.88	0.029	0.44	0.29	0.77	0.59
Valerate	1.72	1.60	1.74	0.083	1.62	1.77	1.57	0.158	0.78	0.89	0.81	0.23
Isovalerate	2.38	2.14	1.87	0.249	1.98	1.80	1.85	0.136	0.17	0.10	0.76	0.53
Acetate:Propionate	3.45	3.18	3.21	0.294	2.95	2.73	2.83	0.262	0.22	0.30	0.28	0.51
NH_3_, mM	6.93	8.08	8.00	0.587	8.69	8.27	8.01	0.422	0.20	0.68	0.51	0.93
Protozoa, cell × 10^3^/mL	627	690	574	70.4	408	457	456	57.2	0.01	0.94	0.29	0.14

^1^ Least square means from 4 Latin squares (12 cows).

**Table 5:** Apparent total-tract digestibility (%) of nutrients in lactating Holstein and Ayrshire cows fed diets formulated to provide 85%, 100%, or 115% of metabolizable protein (MP) requirement ^1^.

	Holstein				Ayrshire							
	MP			SEM	MP			SEM	*p*			
	85%	100%	115%		85%	100%	115%		Breed	MP		Breed × MP
Item										Linear	Quadratic	
DM	67.4	68.1	69.2	0.75	70.1	70.6	70.2	0.77	0.04	0.12	0.75	0.30
OM	69.0	69.9	70.8	0.79	71.7	72.2	72.0	0.75	0.03	0.10	0.82	0.44
CP	61.7	65.9	67.6	1.00	65.6	68.7	69.2	0.86	0.01	<0.01	0.12	0.46
NDF	37.6	39.1	39.6	1.61	44.2	42.0	43.8	1.94	0.05	0.52	0.50	0.34
ADF	36.1	37.8	38.7	2.37	43.6	40.7	41.9	1.51	0.02	0.84	0.63	0.43
Starch	97.5	97.9	98.3	0.29	97.6	98.1	98.3	0.41	0.77	0.04	0.89	0.94
Gross energy	66.7	67.8	68.7	0.86	69.8	70.1	70.0	0.74	0.02	0.09	0.78	0.39

^1^ Least square means from 4 Latin squares (12 cows).

**Table 6:** Enteric methane production of lactating Holstein and Ayrshire cows fed diets formulated to provide 85%, 100%, or 115% of metabolizable protein (MP) requirement ^1^.

	Holstein				Ayrshire							
	MP			SEM	MP			SEM	*p*			
	85%	100%	115%		85%	100%	115%		Breed	MP		Breed × MP
Item										Linear	Quadratic	
DMI, kg/d ^2^	25.5	26.0	26.3	1.13	19.4	20.2	20.9	1.06	<0.01	0.01	0.72	0.53
CH_4_												
g/d	454	487	510	37.3	376	390	391	35.3	0.06	0.03	0.67	0.42
g/kg DMI	17.7	19.1	19.6	1.34	19.1	19.2	18.6	1.34	0.93	0.27	0.59	0.14
g/kg milk ^3^	10.8	11.4	11.9	1.14	12.0	12.3	11.9	1.09	0.66	0.21	0.55	0.30
g/kg ECM ^3^	10.1	10.8	11.3	0.85	10.8	10.9	10.6	0.82	0.94	0.21	0.65	0.20

^1^ Least square means from 4 Latin squares (12 cows) ^2^ Determined for 5 consecutive days in the chambers. ^3^ Yields of milk, ECM, milk fat, and milk protein measured over the 7-d collection periods.

**Table 7:** Energy intake, expenditure and partitioning in lactating Holstein and Ayrshire cows fed diets formulated to provide 85%, 100%, or 115% of metabolizable protein (MP) requirement ^1^.

	Holstein				Ayrshire							
	MP			SEM	MP			SEM	*P*			
	85%	100%	115%		85%	100%	115%		Breed	MP		Breed × MP
Item										Linear	Quadratic	
Intake, Mcal/d												
Gross energy	116	118	117	5.5	91.7	92.5	94.3	4.9	0.01	0.57	0.86	0.84
Digestible energy	77.8	79.8	80.1	3.68	63.9	64.8	66.0	3.19	0.01	0.22	0.80	0.94
ME	69.3	70.2	70.0	3.36	56.9	57.3	58.1	2.74	0.01	0.59	0.91	0.94
NE ^2^	40.0	40.8	40.6	2.00	32.4	33.1	34.0	1.73	0.01	0.24	0.79	0.81
Energy expenditureMcal/d												
Fecal	38.6	37.9	36.7	2.16	27.8	27.8	28.3	1.97	<0.01	0.57	0.99	0.56
Urine	2.28	2.95	3.38	0.148	2.01	2.39	2.67	0.152	0.01	<0.01	0.36	0.15
Methane	6.00	6.44	6.73	0.492	4.96	5.14	5.16	0.465	0.06	0.03	0.67	0.42
Heat ^3^	35.0	35.0	35.2	1.20	28.1	28.1	28.7	1.10	<0.01	0.31	0.56	0.76
Milk	29.7	30.3	30.8	1.56	23.2	24.1	24.8	1.55	0.01	0.03	0.90	0.93
Maintenance ^4^	10.7	10.5	10.7	0.35	9.18	9.18	9.27	0.308	0.01	0.57	0.11	0.48
% GE intake												
Fecal	33.3	32.2	31.3	0.86	30.2	29.9	30.0	0.74	0.02	0.09	0.78	0.39
Urine	2.01	2.55	2.90	0.095	2.19	2.58	2.85	0.117	0.56	<0.01	0.40	0.57
Methane	5.18	5.54	5.66	0.387	5.57	5.55	5.34	0.388	0.96	0.47	0.46	0.14
Heat ^3^	30.7	30.3	30.9	0.82	30.8	30.6	30.5	0.71	0.99	0.99	0.51	0.77
Milk	25.4	25.5	26.4	0.60	25.3	26.1	26.3	0.55	0.82	0.04	0.87	0.75
Maintenance ^4^	9.41	9.09	9.40	0.539	10.10	10.10	9.90	0.478	0.29	0.68	0.64	0.61
Milk energy, % ME	42.8	43.1	44.6	1.39	40.8	42.0	42.7	1.04	0.22	0.10	0.87	0.88

^1^ Least square means from 4 Latin squares (12 cows) ^2^ NE = dietary NE concentration × DMI ^3^ Heat energy calculated using the equation 6.34 in INRA [34]: log_10_ (heat energy) = 1.54 + 0.95 × log_10_ BW + 0.54 × log_10_ DMI%BW ^4^ Maintenance energy calculated according to NRC [14] = 0.080 × BW^0.75^.

**Table 8:** Nitrogen utilization in lactating Holstein and Ayrshire cows fed diets formulated to provide 85%, 100%, or 115% of metabolizable protein (MP) requirement ^1^.

	Holstein				Ayrshire							
	MP			SEM	MP			SEM	*p*			
	85%	100%	115%		85%	100%	115%		Breed	MP		Breed × MP
Item										Linear	Quadratic	
Intake N, g/d	552	654	684	29.1	476	543	594	26.6	0.03	<0.01	0.08	0.46
Fecal N												
g/d	211	224	223	13.4	163	171	183	11.1	0.01	0.08	0.74	0.77
% of N intake	38.3	34.1	32.4	1.00	34.4	31.3	30.7	0.86	0.01	<0.01	0.12	0.46
Urinary N												
g/d	119	186	228	11.4	109	150	182	9.0	0.02	<0.01	0.22	0.07
% of N intake	22.1	28.6	33.3	1.19	22.9	27.8	30.8	0.98	0.33	<0.01	0.35	0.32
Total N excretion												
g/d	329	409	451	22.8	273	321	365	18.7	0.01	<0.01	0.38	0.45
% of N intake	60.4	62.8	65.8	1.66	57.3	59.1	61.5	1.30	0.01	0.01	0.79	0.93
Milk N												
g/d	203	215	211	10.3	165	174	180	10.5	0.03	0.01	0.16	0.33
% of N intake	36.6	32.7	30.9	1.05	34.6	32.2	30.4	0.75	0.39	0.01	0.21	0.41
Urinary PD ^2^, mmol/d												
Allantoin	443	443	395	24.2	328	345	312	25.5	<0.01	0.10	0.14	0.68
Uric acid	53.5	54.2	56.3	5.13	30.7	35.9	33.8	4.60	0.01	0.22	0.48	0.58
Total PD	497	497	451	27.6	359	381	346	28.9	<0.01	0.17	0.16	0.70

^1^ Least square means from 6 Latin squares (12 cows) ^2^ Purine derivatives.

**Table 9:** Prediction of metabolizable protein and metabolizable energy in Ayrshire cows using the experimental diets formulated to Holstein cows.

	MP		
Item	85%	100%	115%
Metabolizable protein			
Requirement, g/d ^1^	2410	2410	2410
Supply, g/d	1951	2245	2604
Supply, % requirement	80.9	93.8	109
Allowable milk, kg/d	26.2	32.8	40.6
Metabolizable energy			
Requirement, Mcal/d ^1^	58.1	58.1	58.0
Supply, Mcal/d	57.6	57.3	58.3
Supply, % requirement	99.4	98.8	100.4
Allowable milk, kg/d	35.7	35.4	36.2

^1^ Requirement when fed breed-specific diet.

## Data Availability

All data of this study are available within the manuscript.
